# Single-nucleotide variants in *TGFB1*, *TGFBR2*, *IL17A*, and *IL17F* immune response genes contribute to follicular lymphoma susceptibility and aggressiveness

**DOI:** 10.1038/s41408-020-00365-4

**Published:** 2020-10-06

**Authors:** Guilherme Rossi Assis-Mendonça, Gustavo Jacob Lourenço, Márcia Torresan Delamain, Vladmir Cláudio Cordeiro de Lima, Gisele Wally Braga Colleoni, Cármino Antônio de Souza, Fernando Augusto Soares, Carmen Silvia Passos Lima, José Vassallo

**Affiliations:** 1 Department of Pathology, Faculty of Medical Sciences, University of Campinas, Campinas, SP Brazil; 2 Laboratory of Investigative and Molecular Pathology, Faculty of Medical Sciences, University of Campinas, Campinas, SP Brazil; 3 Laboratory of Cancer Genetics, Faculty of Medical Sciences, University of Campinas, Campinas, SP Brazil; 4 Hematology and Hemotherapy Center, University of Campinas, Campinas, SP Brazil; 5 Department of Medical Oncology, AC Camargo Cancer Center, São Paulo, SP Brazil; 6 Department of Clinical and Experimental Oncology, Federal University of São Paulo, São Paulo, SP Brazil; 7 Department of Internal Medicine, Faculty of Medical Sciences, University of Campinas, Campinas, SP Brazil; 8 Department of Pathology, Rede D’Or Hospitals, São Paulo, SP Brazil

**Keywords:** Risk factors, B-cell lymphoma, Cancer genetics

Follicular lymphoma (FL) is the most frequent subtype of indolent non-Hodgkin lymphoma (NHL) worldwide, encompassing 35% of cases^1^.

Immune dysregulation is one of the hallmarks of lymphomagenesis^2^, and cytokines act on shifting immune responses. In this setting, disruption of the balance among cytokines may be a key event in the susceptibility to NHL^3^. The major immune pathways encompass Th1 (cellular) response, which is regulated by cytokines such as interleukin 12A (IL12A) and IL2, and T helper type 2 (Th2) (humoral) response, which relies on molecules such as IL10 and transforming growth factor-β (TGFβ)^4,5^. It is also known that TGFβ binds to two receptors (TGFBR1 and TGFBR2) that act as important regulators of downstream effectors^5^. More recently, a novel immune-response pathway dependent on the production of IL-17A and IL-17F, the Th17, was also described^6^.

From the genetic point of view, one mechanism responsible for differential cytokine production is the presence of single-nucleotide variants (SNVs) in coding genes^3^. Some previous reports already addressed the role of SNVs within cytokine genes in the risk and/or prognosis of lymphomas, including mixed NHL cohorts, diffuse large B cell lymphoma and, to a lesser extent, FL^3,7^. However, there are still potentially functional SNVs in key cytokine genes that remain largely unexplored in FL pathogenesis.

We aimed, in the present study, to test whether 16 SNVs in eight immune-response genes (*IL12A*, *IL2*, *IL10*, *TGFB1*, *TGFBR1*, *TGFBR2*, *IL17A*, and *IL17F*) affected FL susceptibility (Supplementary Table 1). We selected SNVs with previous evidence of functionality, either due to the modulation of immune responses or due to previous associations with cancer development or prognosis.

The FL cohort comprised newly diagnosed cases, 71 men and 88 women, followed at the Hematology and Hemotherapy Center of the University of Campinas (Campinas, Brazil) and A.C. Camargo Cancer Center (São Paulo, Brazil), and the control group (blood donors) was composed of 109 men and 110 women from the Hematology and Hemotherapy Center of the University of Campinas. All subjects were unrelated individuals and controls denied any previous neoplasm. The gender distribution between groups was similar, but patients were older than controls (median ages of 56.0 and 50.0 years) (Supplementary Table 2). The study was conducted according to the Declaration of Helsinki and was approved by the institutional ethics committees (# 32177014.3.0000.5404 and # 32177014.3.3001.5432). All subjects signed the informed consent.

We extracted genomic DNA from peripheral blood samples of FL patients, and for genotypic comparisons we also extracted genomic DNA of controls’ peripheral blood samples. DNA was extracted using the lithium chloride technique, and final concentrations were set to 50 ng/µL. Genotyping experiments were performed in pre-designed, personalized genotyping plates from the Taqman Openarray® QuantStudio^TM^ 12K Real-Time PCR System (Life Technologies Inc., Carlsbad, CA, USA).

For all SNVs, the Hardy–Weinberg equilibrium (HWE) was calculated using the *χ*^2^ test; HWE was admitted if *p* > 0.05. The pairwise linkage disequilibrium (LD) analysis in the Haploview 4.2 software was used to ensure that markers were appropriate for inclusion in haplotype estimates. In this setting, both the data (genotypic distributions) and the locus information (name and location of SNVs) files were uploaded to Haploview in the linkage format and processed by the program (https://www.broadinstitute.org/haploview/downloads). The output of these data was an LD plot that expressed the LD coefficient (*D*′) among groups of SNVs. A *D*′ value higher than 0.80 was considered significant for haplotype formation. To assess the role of an SNV or haplotype on FL susceptibility, we performed age-adjusted logistic regression analyses, and risks were expressed using odds ratios (ORs) and 95% confidence intervals (95% CIs).

Control samples were in HWE in all tested loci, except in five of them (*IL2* rs2069762, *TGFBR1* rs334348, *TGFBR2* rs3087465, *IL17A* rs3748067, and *IL17F* rs763780), and patients lacked HWE in loci of four SNVs: *IL12A* rs568408, *IL2* rs2069762, *IL10* rs3024491, and *TGFBR1* rs334348 (Supplementary Table 1). The analyses revealed a strong LD among *IL12A* rs583911, rs568408, and rs485497 (*D*′ = 0.93), and among *IL10* rs3024491, rs1800872, and rs1800890 (*D*′ = 0.92) (Supplementary Fig. 1).

Individuals with *TGFB1* rs1800469 CC or CT genotypes had a higher risk of FL development when compared to those with the TT genotype (OR = 2.13, 95% CI: 1.06–4.28) (Table 1). *TGFB1* rs1800469 CC or CT genotypes were also more common in patients with Ann Arbor stages III or IV than in controls (93.3% vs. 84.5%, *p* = 0.02); carriers of these genotypes were under a 2.63 (95% CI: 1.16–5.97)-fold increased risk of developing aggressive FL than others.

**Table 1:** Single-nucleotide variants in immune-response genes associated with follicular lymphoma susceptibility.

Single-nucleotide variant	Patients, *n* (%)	Controls, *n* (%)	OR (95% CI)	*p* value
***TGFB1*** **rs1800469**				
CC	68 (42.7)	81 (37.0)	2.32 (1.10–4.92)	**0.02**
CT	78 (49.1)	104 (47.5)		
TT	13 (8.2)	34 (15.5)	1.00 (reference)	
CC	68 (42.7)	81 (37.0)	1.30 (0.84–2.02)	0.23
CT + TT	91 (57.3)	138 (63.0)	1.00 (reference)	
CC + CT	146 (91.8)	185 (84.5)	2.13 (1.06–4.28)	**0.03**
TT	13 (8.2)	34 (15.5)	1.00 (reference)	
***TGFBR2*** **rs3087465**^**a**^				
GG	86 (55.5)	89 (41.0)	2.33 (0.88–6.12)	0.08
GA	62 (40.0)	113 (52.1)		
AA	7 (4.5)	15 (6.9)	1.00 (reference)	
GG	86 (55.5)	89 (41.0)	2.10 (1.34–3.29)	**0.01**
GA + AA	69 (44.5)	128 (59.0)	1.00 (reference)	
GG + GA	148 (95.5)	202 (93.1)	1.71 (0.66–4.41)	0.26
AA	7 (4.5)	15 (6.9)	1.00 (reference)	
***IL17A*** **rs3748067**^**a**^				
CC	113 (76.4)	104 (48.8)	0 (0–infinite)	0.99
CT	35 (23.6)	106 (49.8)		
TT	0 (0.0)	3 (1.4)	1.00 (reference)	
CC	113 (76.4)	104 (48.8)	4.10 (2.48–6.75)	**<0.001**
CT + TT	35 (23.6)	109 (51.2)	1.00 (reference)	
CC + CT	148 (100.0)	210 (98.6)	0 (0–infinite)	0.99
TT	0 (0.0)	3 (1.4)	1.00 (reference)	
***IL17F*** **rs763780**^**a**^				
TT	104 (68.4)	90 (42.5)	0 (0–infinite)	0.99
TC	46 (30.3)	122 (57.5)		
CC	2 (1.3)	0 (0.0)	1.00 (reference)	
TT	104 (68.4)	90 (42.5)	3.74 (2.32–6.04)	**<0.001**
TC + CC	48 (31.6)	122 (57.5)	1.00 (reference)	
TT + TC	150 (98.7)	212 (100.0)	0 (0–infinite)	0.99
CC	2 (1.3)	0 (0.0)	1.00 (reference)	

*Rs* reference number. *OR* odds ratio (adjusted only by age using logistic regression estimates), *95% CI* 95% confidence interval. Significant values of *p* are presented in bold letters. ^a^The numbers of patients and controls differed from the total numbers enrolled in study because it was not possible to obtain genotypes in some cases.

In addition, subjects harboring *TGFBR2* GG (OR = 2.10, 95% CI: 1.34–3.29), *IL17A* CC (OR = 4.10, 95% CI: 2.48–6.75), or *IL17F* TT (OR = 3.74, 95% CI: 2.32–6.04) genotypes were under increased risks of developing FL when compared to those with other genotypes (Table 1). The remaining SNVs (Supplementary Table 3) or haplotypes (Supplementary Table 4) did not alter FL susceptibility.

Herein, we showed novel associations between four SNVs and FL risk. First, we found that the “C” allele of *TGFB1* rs1800469 (CC or CT genotypes) was associated with an increased risk of developing FL, like what has already been described for a solid malignancy^8^. It is noteworthy that this association was retained in patients with more aggressive disease (Ann Arbor stages III and IV). It was previously demonstrated that individuals with the TT genotype of rs1800469 produce the highest levels of TGFβ compared to the remaining genotypes^9^. In this setting, subjects harboring the CC or CT genotypes may have a less prominent TGFβ-mediated immunosuppression compared to TT individuals. This, ultimately, may allow the proliferation of lymphocytes, neoplastic transformation, and disease dissemination in CC or CT individuals.

We also demonstrated strong associations between *TGFBR2* GG, *IL17A* CC, and *IL17F* TT genotypes and increased risks of developing FL. Intriguingly, there was a lack of HWE in controls for these three SNVs. Since they were previously implicated on immunological modulation^10–12^, we hypothesize that strong selective pressures could be responsible for this phenomenon. Non-compliance to HWE in controls, for the same SNVs, was also observed in other populations^13,14^.

Furthermore, in one study conducted to investigate the role of SNVs within immune-response genes in NHL, Chen et al.^7^ found interactions between *IL12A* rs568408, body mass index, and susceptibility for FL in females. We could not replicate this result in our population, probably due to differences in study designs, and the presence of different genetic backgrounds from a diverse ancestry in Brazil^15^.

In summary, we present, for the first time, preliminary evidence demonstrating that inherited variants in immune-response genes (*TGFB1* rs1800469, *TGFBR2* rs3087465, *IL17A* rs3748067, and *IL17F* rs763780) alter susceptibility to FL and disease aggressiveness. These findings, following proper validation, may represent easily assessable and potentially targetable biomarkers reflecting immune alterations that lead to FL. We believe that further epidemiological and functional studies regarding these genes and SNVs should be conducted with the purpose of data validation and to better understand the biological role of these variants in FL.

## Supplementary information

Supplementary files

## References

[1] Campo E (2011). The 2008 WHO classification of lymphoid neoplasms and beyond: evolving concepts and practical applications. Blood.

[2] Mori T, Takada R, Watanabe R, Okamoto S, Ikeda Y (2001). T-helper (Th)1/Th2 imbalance in patients with previously untreated B-cell diffuse large cell lymphoma. Cancer Immunol. Immunother..

[3] Lan Q (2006). Cytokine polymorphisms in the Th1/Th2 pathway and susceptibility to non-Hodgkin lymphoma. Blood.

[4] Gollob JA, Schnipper CP, Murphy EA, Ritz J, Frank DA (1999). The functional synergy between IL-12 and IL-2 involves p38 mitogen-activated protein kinase and is associated with the augmentation of STAT serine phosphorylation. J. Immunol..

[5] Principe DR (2014). TGF-β: duality of function between tumor prevention and carcinogenesis. J. Natl Cancer Inst..

[6] Jin W, Dong C (2013). IL-17 cytokines in immunity and inflammation. Emerg. Microbes Infect..

[7] Chen Y (2011). Cytokine polymorphisms in Th1/Th2 pathway genes, body mass index, and risk of non-Hodgkin lymphoma. Blood.

[8] Yang L (2016). Genetic polymorphisms of TGFB1, TGFBR1, SNAI1 and TWIST1 are associated with endometrial cancer susceptibility in Chinese Han women. PLoS ONE.

[9] Grainger DJ (1999). Genetic control of the circulating concentration of transforming growth factor type beta1. Hum. Mol. Genet..

[10] Seijo ER (2001). Identification of genetic alterations in the TGFbeta type II receptor gene promoter. Mutat. Res..

[11] Quan Y (2012). Association between IL17 polymorphisms and risk of cervical cancer in Chinese women. Clin. Dev. Immunol..

[12] Wang L (2012). Association analysis of IL-17A and IL-17F polymorphisms in Chinese Han women with breast cancer. PLoS ONE.

[13] Arisawa T (2012). Genetic polymorphisms of IL17A and pri-microRNA-938, targeting IL17A 30-UTR, influence susceptibility to gastric cancer. Hum. Immunol..

[14] Qinghai Z, Yanying W, Yunfang C, Xukui Z, Xiaoqiao Z (2014). Effect of interleukin-17A and interleukin-17F gene polymorphisms on the risk of gastric cancer in a Chinese population. Gene.

[15] Lins TC, Vieira RG, Abreu BS, Grattapaglia D, Pereira RW (2010). Genetic composition of Brazilian population samples based on a set of twenty-eight ancestry informative SNPs. Am. J. Hum. Biol..

