# Supplementary material for: Single-nucleotide variants in TGFB1, TGFBR2, IL17A, and IL17F immune response genes contribute to follicular lymphoma susceptibility and aggressiveness
Source: Blood Cancer J. 2020 Oct 6;10(10):97. doi: 10.1038/s41408-020-00365-4 (PMC7538580; doi:10.1038/s41408-020-00365-4)
Supplement: Supplementary file 1 — Supplementary files [file 41408_2020_365_MOESM1_ESM.docx]

**Supplementary table 1**. Single nucleotide variants addressed in this study and Hardy-Weinberg equilibrium calculation in patients and controls.

| **Variant** | **Patients** | **Controls** |
| --- | --- | --- |
| ***IL12A* rs568408** | n =151; χ2= 4.85  ***p* = 0.02** | n = 219; χ2 = 0.17  p = 0,67 |
| ***IL12A* rs755004** | n =157; *χ^2^=* 1.75  *p* = 0.18 | n = 219; *χ^2^ =* 0.31  *p* = 0.57 |
| ***IL12A* rs485497** | n = 155; *χ ^2^=* 0.17  *p* = 0.67 | n = 217; *χ^2^ =* 0.32  *p* = 0.56 |
| ***IL12A* rs583911** | n = 151; *χ^2^ =* 0.07  *p* = 0.77 | n=217; *χ^2^=* 3.74  *p* = 0.05 |
| ***IL2* rs2069762** | n = 151; *χ^2^ =* 5.45  ***p* = 0.01** | n = 219; *χ^2^ =* 7.34  ***p* = 0.01** |
| ***IL2* rs6822844** | n = 157; *χ^2^ =* 2.02  *p* = 0.15 | n = 219; *χ^2^ =* 1.10  *p* = 0.29 |
| ***IL10* rs1800872** | n =154; *χ^2^ =* 0.07  *p* = 0.77 | n = 217; *χ^2^ =* 0.01  *p* = 0.09 |
| ***IL10* rs3024491** | n =156; *χ^2^ =* 6.13  ***p* = 0.01** | n = 219; *χ^2^ =* 1.55  *p* = 0.21 |
| ***IL10* rs1800890** | n =155; *χ ^2^=* 0.77  p = 0.37 | n = 219; *χ ^2^=* 0.004  *p* = 0.94 |
| ***TGFB1* rs1800469** | n =159; *χ^2^ =* 2.08  *p* = 0.14 | n = 219; *χ^2^ =* 0.004  *p* = 0.94 |
| ***TGFB1* rs1800471** | n =158; *χ^2^ =* 1.68  p = 0.19 | n = 217; *χ^2^ =* 1.19  *p* = 0.27 |
| ***TGFB1* rs6957** | n =154; *χ^2^ =* 1.91  *p* = 0.16 | n = 219; *χ ^2^=* 1.61  *p* = 0.20 |
| ***TGFBR1* rs334348** | n =146; *χ^2^ =* 8.1  ***p* < 0.01** | n = 212; *χ ^2^=* 40.0  ***p* < 0.01** |
| ***TGFBR2* rs3087465** | n =155; *χ^2^ =* 1.01  *p* = 0.30 | n = 217; *χ^2^ =* 6.91  ***p* < 0.01** |
| ***IL17A* rs3748067** | n =148; *χ^2^ =* 2.66  *p* = 0.10 | n = 213; *χ^2^ =* 17.7  ***p* < 0.01** |
| ***IL17F* rs763780** | n =152; *χ^2^ =* 1.55  *p* = 0.21 | n = 212; *χ^2^ =*34.5  ***p* < 0.01** |

Rs, reference number. Hardy-Weinberg Equilibrium was admitted if p > 0.05. Significant values of *p* are presented in bold letters.

**Supplementary table 2**. Clinicopathological aspects of follicular lymphoma patients and controls.

| **Variable** | **Patients Median (range) or n (%)** | **Controls Median (range) or n (%)** | ***p* value** |
| --- | --- | --- | --- |
| **Age (years)** | 56.0 (20-94) | 50.0 (25-68) | < 0.001 |
| **Gender** |  |  |  |
| Male | 71 (44.7) | 109 (49.8) | 0.32 |
| Female | 88 (55.3) | 110 (50.2) |  |
| **B symptoms** |  |  |  |
| Present | 57 (35.8) | NA | NA |
| Absent | 100 (62.9) | NA | NA |
| Not available | 2 (1.3) | NA | NA |
| **Bulky disease** |  |  |  |
| Present | 40 (25.2) | NA | NA |
| Absent | 109 (68.5) | NA | NA |
| Not available | 10 (6.3) | NA | NA |
| **Bone marrow infiltration** |  |  |  |
| Present | 71 (44.7) | NA | NA |
| Absent | 88 (55.3) | NA | NA |
| **Tumor stage*** |  |  |  |
| I | 17 (10.7) | NA | NA |
| II | 17 (10.7) | NA | NA |
| III | 41 (25.8) | NA | NA |
| IV | 84 (52.8) | NA | NA |

NA, Not applicable. *Tumor stage was defined using the Ann Arbor classification. Significant values of *p* are presented in bold letters.

**Supplementary table 3**. Variants in immune response genes without roles in follicular lymphoma susceptibility.

| **Variant** | **Patients n (%)** | **Controls n (%)** | **OR* (95% CI)** | ***p* value** |
| --- | --- | --- | --- | --- |
| ***IL12A* rs568408*** |  |  |  |  |
| GG | 112 (74.2) | 169 (77.2) | 1.00 (reference) |  |
| GA | 32 (21.2) | 46 (21.0) |  |  |
| AA | 7 (4.6) | 4 (1.8) | 2.73 (0.72-10.29) | 0.13 |
| GG | 112 (74.2) | 169 (77.2) | 1.00 (reference) |  |
| GA+AA | 39 (25.8) | 50 (22.8) | 1.19 (0.71-1.97) | 0.14 |
| GG+GA | 144 (95.4) | 215 (98.2) | 1.00 (reference) |  |
| AA | 7 (4.6) | 4 (1.8) | 2.67 (0.72-9.89) | 0.14 |
| ***IL12A* rs755004*** |  |  |  |  |
| GG | 127 (80.9) | 163 (74.4) | 1.00 (reference) |  |
| GA | 30 (19.1) | 53 (24.2) |  |  |
| AA | 0 (0.0) | 3 (1.4) | 0 (0-infinite) | 0.99 |
| GG | 127 (80.9) | 163 (74.4) | 1.00 (reference) |  |
| GA+AA | 30 (19.1) | 56 (25.6) | 0.73 (0.43-1.24) | 0.25 |
| GG+GA | 157 (100.0) | 216 (98.6) | 1.00 (reference) |  |
| AA | 0 (0.0) | 3 (1.4) | 0 (0-infinite) | 0.99 |
| ***IL12A* rs485497*** |  |  |  |  |
| AA | 40 (25.8) | 61 (28.1) | 1.00 (reference) |  |
| AG | 80 (51.6) | 112 (51.6) |  |  |
| GG | 35 (22.6) | 44 (20.3) | 1.47 (0.75-2.88) | 0.25 |
| AA | 40 (25.8) | 61 (28.1) | 1.00 (reference) |  |
| AG+GG | 115 (74.2) | 156 (71.9) | 1.31 (0.79-2.16) | 0.28 |
| AA+AG | 120 (77.5) | 173 (79.8) | 1.00 (reference) |  |
| GG | 35 (22.6) | 44 (20.3) | 1.27 (0.74-2.17) | 0.37 |
| ***IL12A* rs583911*** |  |  |  |  |
| AA | 47 (31.1) | 56 (25.8) | 1.00 (reference) |  |
| AG | 76 (50.3) | 122 (56.2) |  |  |
| GG | 28 (18.6) | 39 (18.0) | 0.75 (0.38-1.48) | 0.40 |
| AA | 47 (31.1) | 56 (25.8) | 1.00 (reference) |  |
| AG+GG | 104 (68.9) | 161 (74.2) | 0.71 (0.44-1.16) | 0.17 |
| AA+AG | 123 (81.4) | 178 (82.0) | 1.00 (reference) |  |
| GG | 28 (18.5) | 39 (18.0) | 0.91 (0.51-1.62) | 0.75 |
| ***IL2* rs2069762*** |  |  |  |  |
| TT | 84 (55.6) | 109 (49.8) | 1.00 (reference) |  |
| TG | 64 (42.4) | 102 (46.6) |  |  |
| GG | 3 (2.0) | 8 (3.6) | 0.53 (0.13-2.15) | 0.37 |
| TT | 84 (55.6) | 109 (49.8) | 1.00 (reference) |  |
| TG+GG | 67 (44.4) | 110 (50.2) | 0.78 (0.50-1.21) | 0.28 |
| TT+TG | 148 (98.0) | 211 (96.4) | 1.00 (reference) |  |
| GG | 3 (2.0) | 8 (3.6) | 0.57 (0.14-2.27) | 0.43 |
| ***IL2* rs6822844*** |  |  |  |  |
| GG | 125 (79.6) | 183 (83.5) | 1.00 (reference) |  |
| GT | 32 (20.4) | 33 (15.1) |  |  |
| TT | 0 (0.0) | 3 (1.4) | 0 (0-infinite) | 0.99 |
| GG | 125 (79.6) | 183 (83.5) | 1.00 (reference) |  |
| GT+TT | 32 (20.4) | 36 (16.5) | 1.29 (0.74-2.27) | 0.36 |
| GG+GT | 157 (100.0) | 216 (98.6) | 1.00 (reference) |  |
| TT | 0 (0.0) | 3 (1.4) | 0 (0-infinite) | 0.99 |
| ***IL10* rs1800872*** |  |  |  |  |
| CC | 69 (44.8) | 90 (41.5) | 1.00 (reference) |  |
| CA | 67 (43.5) | 100 (46.1) |  |  |
| AA | 18 (11.7) | 27 (12.4) | 0.75 (0.36-1.53) | 0.43 |
| CC | 69 (44.8) | 90 (41.5) | 1.00 (reference) |  |
| CA+AA | 85 (55.2) | 127 (58.5) | 0.78 (0.50-1.21) | 0.27 |
| CC+CA | 136 (88.3) | 190 (87.6) | 1.00 (reference) |  |
| AA | 18 (11.7) | 27 (12.4) | 0.86 (0.44-1.67) | 0.65 |
| ***IL10* rs3024491*** |  |  |  |  |
| CC | 78 (50.0) | 103 (47.0) | 1.00 (reference) |  |
| CA | 73 (46.8) | 100 (45.7) |  |  |
| AA | 5 (3.2) | 16 (7.3) | 0.51 (0.16-1.61) | 0.25 |
| CC | 78 (50.0) | 103 (47.0) | 1.00 (reference) |  |
| CA+AA | 78 (50.0) | 116 (53.0) | 1.01 (0.65-1.56) | 0.96 |
| CC+CA | 151 (96.8) | 203 (92.7) | 1.00 (reference) |  |
| AA | 5 (3.2) | 16 (7.3) | 0.49 (0.16-1.45) | 0.20 |
| ***IL10* rs1800890*** |  |  |  |  |
| TT | 95 (61.3) | 126 (57.5) | 1.00 (reference) |  |
| TA | 55 (35.5) | 80 (36.6) |  |  |
| AA | 5 (3.2) | 13 (5.9) | 0.64 (0.20-2.0) | 0.44 |
| TT | 95 (61.3) | 126 (57.5) | 1.00 (reference) |  |
| TA+AA | 60 (38.7) | 93 (42.5) | 0.91 (0.59-1.42) | 0.70 |
| TT+TA | 150 (96.7) | 206 (94.1) | 1.00 (reference) |  |
| AA | 5 (3.3) | 13 (5.9) | 0.64 (0.21-1.95) | 0.44 |
| ***TGFB1* rs1800471*** |  |  |  |  |
| GG | 145 (91.8) | 187 (86.2) | 1.00 (reference) |  |
| GC | 12 (7.6) | 30 (13.8) |  |  |
| CC | 1 (0.6) | 0 (0.0) | Infinite (0-infinite) | 0.99 |
| GG | 145 (92.3) | 187 (86.2) | 1.00 (reference) |  |
| GC+CC | 12 (7.7) | 30 (13.8) | 0.59 (0.28-1.25) | 0.17 |
| GG+GC | 157 (99.4) | 217 (100.0) | 1.00 (reference) |  |
| CC | 1 (0.6) | 0 (0.0) | Infinite (0-infinite) | 0.99 |
| ***TGFB1* rs6957*** |  |  |  |  |
| AA | 93 (60.4) | 136 (62.1) | 1.00 (reference) |  |
| AG | 57 (37.0) | 77 (35.1) |  |  |
| GG | 4 (2.6) | 6 (2.8) | 1.02 (0.27-3.92) | 0.96 |
| AA | 93 (60.4) | 136 (62.1) | 1.00 (reference) |  |
| AG+GG | 61 (39.6) | 83 (37.9) | 1.19 (0.76-1.87) | 0.44 |
| AA+AG | 150 (97.4) | 213 (97.3) | 1.00 (reference) |  |
| GG | 4 (2.6) | 6 (2.7) | 0.96 (0.25-3.72) | 0.96 |
| ***TGFBR1* rs334348*** |  |  |  |  |
| AA | 64 (43.8) | 74 (34.9) | 1.00 (reference) |  |
| AG | 76 (52.1) | 135 (63.7) |  |  |
| GG | 6 (4.1) | 3 (1.4) | 3.54 (0.75-16.55) | 0.10 |
| AA | 64 (43.8) | 74 (34.9) | 1.00 (reference) |  |
| AG+GG | 82 (56.2) | 138 (65.1) | 0.69 (0.43-1.08) | 0.11 |
| AA+AG | 140 (95.9) | 209 (98.6) | 1.00 (reference) |  |
| GG | 6 (4.1) | 3 (1.4) | 4.25 (0.96-18.74) | 0.05 |

Rs, reference number; OR*, odds ratio adjusted only by age using logistic regression estimates; 95% CI, 95% confidence interval; *, the numbers of patients and or controls differed from the total numbers enrolled in study because it was not possible to obtain genotypes in some cases.

| **Supplementary table 4**. Haplotypes in *IL10* and *IL12A* and follicular lymphoma susceptibility. | | | | |
| --- | --- | --- | --- | --- |
| **Haplotypes (%)** | **Patients n (%)** | **Controls n (%)** | **OR* (95% CI)** | ***p* value** |
| *IL12A* GGG | 68 (45.0) | 101 (46.5) | 0.94 (0.62-1.43) | 0.79 |
| Other haplotypes | 83 (55.0) | 116 (53.5) | 1.00 (reference) |  |
| *IL12A* GAG | 25 (16.4) | 27 (12.4) | 1.40 (0.77-2.52) | 0.26 |
| Other haplotypes | 127 (83.6) | 191 (87.6) | 1.00 (reference) |  |
| *IL12A* GAA | 24 (15.8) | 26 (12.5) | 1.39 (0.76-2.53) | 0.27 |
| Other haplotypes | 128 (84.2) | 192 (87.5) | 1.00 (reference) |  |
| *IL12A* GGA | 99 (65.5) | 154 (71.6) | 0.74 (0.47-1.17) | 0.20 |
| Other haplotypes | 52 (34.5) | 61 (28.4) | 1.00 (reference) |  |
| *IL12A* AGG | 103 (68.2) | 152 (70.0) | 0.92 (0.59-1.45) | 0.74 |
| Other haplotypes | 48 (31.8) | 65 (30.0) | 1.00 (reference) |  |
| *IL12A* AAG | 39 (25.6) | 49 (22.5) | 1.20 (0.74-1.95) | 0.44 |
| Other haplotypes | 113 (74.4) | 169 (77.5) | 1.00 (reference) |  |
| *IL10* CCT | 128 (81.5) | 174 (79.8) | 1.12 (0.66-1.89) | 0.66 |
| Other haplotypes | 29 (18.5) | 44 (20.2) | 1.00 (reference) |  |
| *IL10* CAT | 82 (53.9) | 127 (58.5) | 0.82 (0.54-1.25) | 0.37 |
| Other haplotypes | 70 (46.1) | 90 (41.5) | 1.00 (reference) |  |
| *IL10* ACA | 56 (35.9) | 90 (41.1) | 0.80 (0.52-1.22) | 0.30 |
| Other haplotypes | 100 (64.1) | 129 (58.9) | 1.00 (reference) |  |

OR*, odds ratio adjusted only by age using logistic regression estimates; 95% CI, 95% confidence interval.


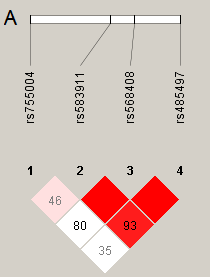


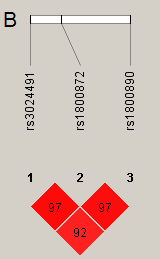


**Supplementary figure 1**. Linkage disequilibrium plots in the study population, considering (**A**) *IL12A* and (**B**) *IL10*. The numbers within the squares represent the estimates of LD (D’) between/among groups of single nucleotide variants. The squares highlighted in red have significant LD index (higher than 0.8). Red squares without numbers represent a D’ value of 100.
